# Genetic Analysis of the *HSPA1A*, *HSPA1B*, and *HSPA1L* Genes in Patients with Schizophrenia from Taiwan

**DOI:** 10.3390/genes17070727

**Published:** 2026-06-23

**Authors:** Ying-Chieh Wang, Shih-Hsin Hsu, Hsin-Yao Tsai, Min-Chih Cheng

**Affiliations:** Department of Psychiatry, Yuli Branch, Taipei Veterans General Hospital, Hualien 98142, Taiwan; psylab2@mail2000.com.tw (Y.-C.W.); filvhsu@gmail.com (S.-H.H.); ashleytsai0808@gmail.com (H.-Y.T.)

**Keywords:** schizophrenia, heat shock protein 70, mutation detection, rare mutation

## Abstract

**Background/Objectives**: The genes encoding *HSPA1A*, *HSPA1B*, and *HSPA1L*, located in the MHC class III region at 6p21.3–22.1, a region implicated in susceptibility to schizophrenia, are critical regulators of neurodevelopmental processes and contribute to synaptic neuroprotection. This study investigated whether the *HSPA1A*, *HSPA1B*, and *HSPA1L* genes are associated with schizophrenia. **Methods**: We sequenced the coding regions of *HSPA1A*, *HSPA1B*, and *HSPA1L* from 100 patients with schizophrenia to identify genetic variants. Further, we conducted a genetic association analysis of three SNPs (rs9469057, rs142416335, and rs2075800) in the *HSPA1L* gene in 519 patients with schizophrenia and 1492 healthy controls from the Taiwan Biobank. We analyzed the function of the HSPA1L protein via immunoblotting. **Results**: We identified 17 coding variants, including 8 missense and 9 synonymous mutations, in 100 patients with schizophrenia. Three variants (*HSPA1L*^p.Ala8Pro^, *HSPA1L*^p.Ala8Thr^, and *HSPA1L*^p.Glu602Lys^) in the *HSPA1L* gene did not exhibit any significant differences in allele or genotype frequencies between patients and control subjects. Notably, one ultra-rare missense mutation, *HSPA1L*^p.Val262Met^, was not documented in the control sample in Taiwan BioBank. Immunoblotting revealed *HSPA1L*^p.Val262Met^ mutant with decreased protein expression in SH-SY5Y cells compared with the wild type. **Conclusions**: While common variants in the *HSPA1A*, *HSPA1B*, and *HSPA1L* genes do not seem to be significant genetic risk factors for schizophrenia in this cohort, the ultra-rare mutation, *HSPA1L*^p.Val262Met^, significantly reduces protein expression. These preliminary findings suggest that a potential loss-of-function or reduced expression of the *HSPA1L* gene may be a predisposing factor contributing to schizophrenia vulnerability in certain individuals. However, the finding should be replicated in other independent samples. The in vitro and in vivo impacts of the associated mutation at the *HSPA1L* gene on the pathophysiology of schizophrenia are worthy of future investigation.

## 1. Introduction

Schizophrenia is a complex, chronic neuropsychiatric disorder characterized by distortions in thinking, perception, emotions, and behavior [1]. The etiology of schizophrenia is highly heritable, with estimates suggesting that genetics accounts for up to 80% of the risk [2]. A significant amount of evidence indicates that schizophrenia is a disorder related to synaptic connectivity, neurodevelopment, and neuroinflammation [3,4].

The heat shock protein 70 (HSP70) family plays several roles, including regulating stress responses, stabilizing existing proteins to prevent aggregation, managing the folding of newly translated proteins, and supporting cytoprotective and immunomodulatory functions, as well as cellular repair mechanisms [5,6,7,8]. A growing body of evidence indicates that HSP70 proteins are critical regulators of neurodevelopmental processes and contribute to synaptic neuroprotection [9,10]. In addition, stimuli that trigger the stress response and induce HSP70 synthesis activate memory processes and the formation of new synapses [11]. It has been hypothesized that aberrant expression of HSP70 may be linked to structural brain abnormalities observed in schizophrenic patients [12]. Hence, the genes encoding HSP70 family proteins might be involved in the neurodevelopmental mechanism of schizophrenia and could represent candidates for schizophrenia therapy.

The *HSPA1A*, *HSPA1B*, and *HSPA1L* genes encode 70-kDa heat shock proteins, which are members of the HSP70 family [5]. Our goal is to determine whether these three genes contribute to the genetic risk of schizophrenia for several reasons. First, the *HSPA1A*, *HSPA1B*, and *HSPA1L* genes are located in the major histocompatibility complex class III region at 6p21.3-22.1, a region implicated in susceptibility to schizophrenia [13,14]. Second, studies have identified elevated levels of antibodies against HSP70 in patients with schizophrenia [15,16]. Third, polymorphisms in the *HSPA1A*, *HSPA1B*, and *HSPA1L* genes have been associated with schizophrenia across multiple populations of diverse ethnic backgrounds [17,18,19,20,21,22]. Finally, genes associated with activity-regulated cytoskeletal-associated protein (ARC) may contribute to synaptic dysfunction in the pathogenesis of schizophrenia [23,24]. RNA sequencing results indicated that the *HSPA1A*, *HSPA1B*, and *HSPA1L* genes were significantly downregulated in isogenic human ARC-knockout HEK293 cells, suggesting that HSP70 proteins may interact with ARC and could be related to the pathophysiology of schizophrenia [25]. Taken together, we propose that the paradox of systemic immune-related hyperreactivity versus intrinsic synaptic down-regulation and proteostatic deficits suggests that genetic disruptions in the HSP70 chaperone architecture weaken essential neuroprotective boundaries, increasing the risk of developing schizophrenia.

We investigated whether HSP70 family genes were associated with schizophrenia by resequencing the protein-coding regions of the *HSPA1A*, *HSPA1B*, and *HSPA1L* genes in a sample of patients with schizophrenia from Taiwan.

## 2. Materials and Methods

### 2.1. Subjects

A total of 100 schizophrenic patients (48 males, 52 females, mean age = 45 ± 11 years) were used for screening for mutations. An independent sample set comprised 519 schizophrenic patients (254 males, mean age = 43 ± 9 years; 265 females, mean age = 49 ± 10 years) was used for the association study. Schizophrenic patients fulfilling the DSM-5 diagnostic criteria for schizophrenia were recruited in this study. Patients with organic brain syndromes, intellectual disabilities, substance-related psychosis, or mood disorders featuring psychosis were excluded. All the patients were Han Chinese from Taiwan. The ethics committee of the Antai-Tian-Sheng Memorial Hospital Institutional Review Board approved the study (Approval No. 101020), and written informed consent was obtained after the procedures were fully explained. Genomic DNA had been isolated from peripheral blood using the Gentra Puregene Blood Kit (QIAGEN, Germantown, MD, USA).

### 2.2. Bioinformatics of the HSPA1A, HSPA1B and HSPA1L Genes and In Silico Analysis

Information on the *HSPA1A*, *HSPA1B*, and *HSPA1L* genes was obtained from the NCBI database (http://www.ncbi.nlm.nih.gov, accessed on 1 May 2026) and the UCSC Genome Bioinformatics website (http://genome.ucsc.edu/index.html, accessed on 1 May 2026). The missense mutations were analyzed using the following publicly available prediction programs: Polyphen-2, SIFT, and CADD. It was assessed whether the identified mutations were documented in the dbSNP (https://www.ncbi.nlm.nih.gov/snp/, accessed on 1 May 2026), gnomAD dataset (https://gnomad.broadinstitute.org/, accessed on 1 May 2026), and Taiwan BioBank (https://taiwanview.twbiobank.org.tw, accessed on 15 June 2026).

### 2.3. PCR-Based Sequencing

The exons of the *HSPA1A*, *HSPA1B*, and *HSPA1L* genes from 100 patients with schizophrenia were PCR-amplified and subjected to mutation screening by direct PCR-based sequencing. Optimal PCR primer sequences were generated for each exon using the Primer3 website (https://primer3.ut.ee/, accessed on 1 April 2025). Primer sequences, optimal annealing temperatures, and amplicon sizes are listed in Supplementary Table S1. Genomic DNA (75 ng) was amplified in a reaction volume of 15 μL, which included 0.5 μM of each forward and reverse primer, 0.4 mM of dNTPs, 1× PCR buffer for KOD FX, and 1 U KOD FX DNA polymerase (TOYOBO Co., Ltd., Osaka, Japan). The PCR cycling conditions included an initial pre-denaturation step at 94 °C for 2 min. This was followed by 25 cycles consisting of denaturation at 98 °C for 10 s, annealing at the optimal temperature for each amplicon for 30 s, and extension at 68 °C for 1 min for each kilobase. After amplification, aliquots of the PCR products were processed using an illustra^TM^ ExoProStar^TM^ 1-Step Kit (Cytiva, Buckinghamshire, UK) according to the manufacturer’s instructions to remove residual primers and dNTPs. The purified PCR products were sequenced using an ABI Prism™ BigDye™ Terminator Cycle Sequencing Ready Reaction Kit (version 3.1) on a SeqStudio™ Genetic Analyzer (Thermo Fisher Scientific Inc., Waltham, MA, USA), following the manufacturer’s instructions. The quality of sequencing was assessed using SeqStudio™ Genetic Analyzer software v1.2.3 according to the manufacturer’s protocol. Repeat PCR and sequencing were performed to verify all variants in both directions.

### 2.4. Genetic Association Analysis

This study conducted a genetic association analysis of three SNPs from the *HSPA1L* gene: rs9469057, rs142416335, and rs2075800. The analysis involved 519 patients diagnosed with schizophrenia and 1492 healthy controls from the Taiwan Biobank. Genotyping was performed using the QuantStudio 3 Real-Time PCR System in conjunction with the TaqMan™ SNP Genotyping Assay (Thermo Fisher Scientific, Waltham, MA, USA). Since rs9469057 is a triallelic SNP, genotyping for this variant utilized two pre-designed TaqMan assays. The context sequence for rs9469057 is GTGGTGCCCAGGTCGATGCCTATGG[C/G/T]GATTCCCTTGGCAGTAGCCATGGTT. The context sequences for rs142416335 and rs2075800 were TCGCAGGCGGTGCGCAGCCGCCTCA[C/T]GGCTCGCTTGTTCTGGCTGATGTCC and GTGATGATAGGGTTACACATCTGCT[C/T]CAATTCCTTTCTCTTATGATCAAAC. PCR reactions were conducted in a total volume of 10 μL, which included 5 ng of genomic DNA, 0.25 μL of 40× pre-designed TaqMan™ SNP Genotyping Assay (comprising two unlabeled primers and VIC/FAM dye-labeled MGB probes), and 5 μL of 2× TaqManR GTXpress™ Master Mix. The thermal cycling protocol began with initial denaturation at 95 °C for 2 min, followed by 40 cycles of denaturation at 95 °C for 15 s and 60 °C for 40 s. Finally, genotype calls were determined from allelic discrimination plots generated by QuantStudio™ 3 software, which used fluorescence signal intensity to differentiate among three genotypes. Differences in SNP allele and genotype frequencies between the two groups were assessed using a chi-squared test. *p*-values less than 0.05 were considered statistically significant. Allelic odds ratios (OR) and their 95% confidence intervals (CI) for rs9469057, rs142416335, and rs2075800 were calculated based on the allele frequencies.

### 2.5. Gene Constructs and Transient Transfection of Cell Lines, and Immunoblotting

The cDNA encoding the *HSPA1L* gene was synthesized by MDBio, Inc., Taiwan, and subcloned into the pCMV6-Myc-DDK-tagged and pCMV6-AC-tGFP-tagged vectors (Origene Technologies, Inc., Rockville, MD, USA) using the SgfI and MluI enzymes. The mutant-type plasmids were generated using the QuikChange^®^ Lightning Site-Directed Mutagenesis Kit (Agilent Technologies, Santa Clara, CA, USA) according to the manufacturer’s instructions. The authenticity of plasmid sequences was verified by qualitative restriction enzyme digestion and fluorescence-based cycle sequencing. The human neuroblastoma SH-SY5Y cell line (ECACC-94030304, Merck, Germany) was cultured in Dulbecco’s Modified Eagle’s Medium (DMEM) supplemented with 10% fetal bovine serum and 1% antibiotics at 37 °C in a 5% CO_2_ environment. The cells were transfected with constructs using Lipofectamine™ 3000 (Invitrogen, Waltham, MA, USA) and cultured in 24-well plates for immunoblot analysis. Immunoblotting was performed according to standard protocols using the primary antibodies listed below. Rat anti-DYKDDDDK Tag (MCA4764GA, Bio-Rad Laboratories Inc., Hercules, CA, USA), mouse anti-turboGFP (TA150041; Origene Technologies, Inc., Rockville, MD, USA), and mouse anti-GAPDH (G8795; Sigma-Aldrich, Saint Louis, MO, USA). Horseradish peroxidase-conjugated goat anti-rat IgG (NA935V, Cytiva, Buckinghamshire, UK) and human anti-mouse IgG (5220-0341; SeraCare KPL, Milford, MA, USA) were used as secondary antibodies. Chemiluminescence was visualized using an enhanced chemiluminescence detection system (GTX400006; GeneTex, Hsinchu City, Taiwan). All tests were conducted in triplicate. Statistically significant differences between the two groups were assessed using a *p*-value threshold of below 0.05.

## 3. Results

### 3.1. Mutation Detection the HSPA1A, HSPA1B, and HSPA1L Genes

We initially screened for mutations of the *HSPA1A*, *HSPA1B*, and *HSPA1L* genes in 100 patients with schizophrenia using PCR-based sequencing. We identified a total of 17 coding variants, which included 8 missense mutations and 9 synonymous mutations (Table 1). Two specific mutations (*HSPA1A*^p.Glu100Asp^ and *HSPA1L*^p.Val262Met^) were not found in the control sample from the Taiwan BioBank. The *HSPA1L*^p.Val262Met^ mutation (rs142416335) exhibited a minor allele frequency (MAF) of less than 0.1% in both the 1000 Genomes Project and gnomAD databases. Sequence electropherogram of *HSPA1L*^p.Val262Met^ mutation is shown in Supplementary Figure S1. In silico analysis indicated that three mutations (*HSPA1L*^p.Ala8Pro^, *HSPA1L*^p.Ala8Thr^, and *HSPA1L*^p.Glu602Lys^) were predicted to be pathogenic by Polyphen-2 and SIFT analyses (Table 1).

### 3.2. Association Study

Taiwan Biobank (https://taiwanview.twbiobank.org.tw/, accessed on 15 June 2026) is a nationwide research database that collects, stores, and analyzes biological data necessary for research aimed at tracing common chronic diseases occurring locally in Taiwan. We conducted a genetic association analysis of four SNPs (*HSPA1L*^p.Ala8Pro^, *HSPA1L*^p.Ala8Thr^, *HSPA1L*^p.Val262Met^, and *HSPA1L*^p.Glu602Lys^) in the *HSPA1L* gene in 519 patients with schizophrenia and 1492 healthy controls from the Taiwan Biobank. Notably, *HSPA1L*^p.Val262Met^ was not documented in the control sample from Taiwan BioBank and the odds ratio was 37.65. The other three SNPs (*HSPA1L*^p.Ala8Pro^, *HSPA1L*^p.Ala8Thr^, and *HSPA1L*^p.Glu602Lys^) did not exhibit any significant differences in allele or genotype frequencies between patients and control subjects (Table 2).

### 3.3. Immunoblotting Assay

**Figure 1:**
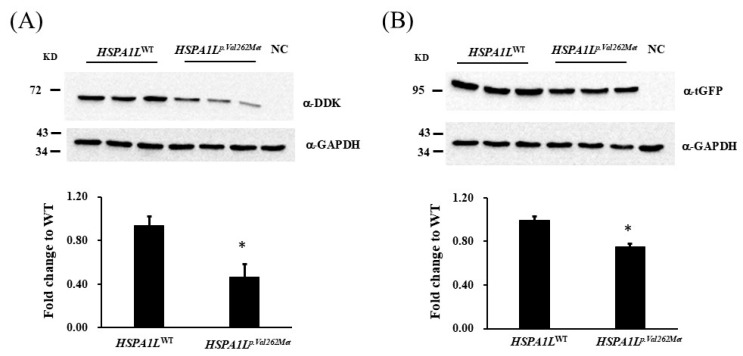
Immunoblotting confirmed HSPA1L protein overexpression in lysates using anti-DDK (**A**) or anti-tGFP (**B**) antibodies. The lysates were analyzed in parallel by anti-GAPDH immunoblotting (*n* = 3) for normalization. WT, wild type. NC, negative control. Graphs represent means ± SD. * *p* < 0.05.

We investigated whether *HSPA1L*^p.Val262Met^ mutation affected HSPA1L protein expression, as determined by immunoblotting of SH-SY5Y cells after 24 h of transient expression of the mutant or WT protein. HSPA1L-DDK and HSPA1L-tGFP fusion protein expression significantly decreased in SH-SY5Y cells carrying the *HSPA1L*^p.Val262Met^ mutant compared with *HSPA1L*^WT^ (Figure 1). The steady-state protein expression levels of the *HSPA1L*^p.Val262Met^ mutant showed a more pronounced downregulation when fused to the DDK tag compared to the tGFP tag.

## 4. Discussion

In the present study, we evaluated the genetic contribution of *HSPA1A*, *HSPA1B*, and *HSPA1L* to schizophrenia risk in a Taiwanese cohort and identified an ultra-rare *HSPA1L*^p.Val262Met^ mutation. The *HSPA1L*^p.Val262Met^ mutation was absent in 1492 healthy controls from the local Taiwan BioBank database and had a global MAF of less than 0.0001 in gnomAD, making it a priority for functional characterization.

The common variant hypothesis suggests that schizophrenia results from the cumulative impact of numerous common genetic variants, each contributing a small to moderate risk of developing the disorder [26,27]. In contrast, the rare mutation hypothesis proposes that schizophrenia is caused by rare mutations that significantly affect multiple genes across individuals [28,29]. Prior candidate gene studies have reported significant associations between common SNPs in the *HSPA1A*, *HSPA1B*, and *HSPA1L* genes and schizophrenia in Korean and Polish populations [17,18,19,21]. However, we sequenced these three genes in patients with schizophrenia from Taiwan, and further analysis showed no association between SNPs in *HSPA1A*, *HSPA1B*, and *HSPA1L* and schizophrenia. The lack of a significant association with common variants in the *HSPA1A*, *HSPA1B*, and *HSPA1L* genes in this Taiwanese cohort likely reflects underlying ethnic differences, population substructure, varying linkage disequilibrium patterns, and differences in clinical assessment [30]. Thus, common variants typically confer very small individual risks, which may require substantially larger sample sizes to detect reliably. Conversely, our discovery of the *HSPA1L*^p.Val262Met^ mutation aligns with the emerging consensus that rare, highly penetrant genetic variations also play a critical role in the complex genetic architecture of schizophrenia.

HSP70 family proteins function as essential molecular chaperones that stabilize newly translated proteins, prevent misfolding and aggregation, and provide cytoprotection during stress [5,6]. A deficit in HSPA1L function could predispose individuals to schizophrenia through several intersecting neurobiological pathways. For example, HSP70 proteins are continuously synthesized in the nervous system, where they localize to synaptic junctions and postsynaptic densities [10,11]. At the synapse, HSP70 acts as a critical regulator of neuroplasticity, facilitating the folding and transport of synaptic proteins, maintaining synaptic transmission under stress, and supporting memory formation. A reduction in HSPA1L could severely impair these neuroprotective processes, leaving synapses vulnerable to damage or excessive microglial elimination [10]. In the current study, we observed that the expression of HSPA1L-DDK and HSPA1L-tGFP fusion proteins was significantly reduced in SH-SY5Y cells carrying the *HSPA1L*^p.Val262Met^ mutant compared to the *HSPA1L*^WT^. The steady-state protein expression levels of the *HSPA1L*^p.Val262Met^ mutant showed a more significant downregulation when fused to the DDK tag compared to the tGFP tag. This difference in relative fold change between the two tagged constructs is likely due to substantial variations in the biophysical properties and molecular weights of the tags. The marked decrease in steady-state protein abundance suggests that the *HSPA1L*^p.Val262Met^ mutation may lead to altered protein instability or increased degradation. Given that HSPA1L is a molecular chaperone involved in protein folding and cellular stress responses, a significant reduction in its expression could impair proteostasis, potentially contributing to the neurodevelopmental or neurodegenerative pathways associated with schizophrenia.

In addition to its role as a chaperone, HSP70 serves as a powerful anti-inflammatory modulator. For instance, studies have shown that overexpression of HSP70 can suppress neuroinflammatory responses in astrocytes by downregulating pro-inflammatory cytokines such as TNF-α and IL-1, as well as inhibiting COX-2 and iNOS expression [31]. A decrease in the expression of HSPA1L could weaken this anti-inflammatory mechanism, potentially worsening neuroinflammation and making the brain more vulnerable to the inflammatory factors identified as environmental risk factors for schizophrenia. There is evidence linking neuroinflammation—driven by the activation of microglia and astrocytes—to the pathophysiology of schizophrenia [4,31]. While our findings support a functional consequence of the variant, *HSPA1L*^p.Val262Met^, through reduced steady-state protein abundance in vitro, they do not directly establish definitive loss-of-function or haploinsufficiency. Consequently, reduced levels of HSPA1L may impair this anti-inflammatory protection, increasing the brain’s susceptibility to the inflammatory insults, which are recognized as environmental contributors to schizophrenia. These findings suggest that alterations in the structure or function of the HSPA1L protein could play a role in the pathophysiology of schizophrenia in affected individuals.

HSP70 proteins are composed of two primary functional regions: the N-terminal nucleotide binding domain (NBD), also referred to as the ATPase domain, which possesses ATPase activity, and the C-terminal substrate binding domain (SBD), also known as the peptide-binding domain, where client or substrate proteins attach [32]. The *HSPA1L*^p.Val262Met^ mutation is located in the NBD of the HSPA1L protein and *HSPA1L*^p.Val262^ is conserved in several human HSP70 family members [8,32]. We speculated that this mutation may affect ATP binding and hydrolysis. However, further investigation is required to reveal the consequences of the *HSPA1L*^p.Val262Met^ mutation on protein function and the pathogenic contributions of schizophrenia.

Several limitations should be considered when interpreting our findings. First, the sizes of our discovery sample (*n* = 100) and follow-up association cohort (*n* = 519) are relatively small, which restricts our ability to identify additional ultra-rare mutations. The MAFs for SNPs *HSPA1L*^p.Ala8Pro^ and *HSPA1L*^p.Ala8Thr^ are low, ranging from approximately 1.2% to 2.9%. To achieve 80% power at a standard alpha of 0.05 for alleles with about a 2% frequency and an expected OR of 1.5, we would typically require tens of thousands of samples. Second, the control group from the Taiwan BioBank was part of a general population cohort and was not specifically screened for psychiatric conditions using structured clinical interviews. As a result, there may be an approximate 1% bias, which aligns with the baseline prevalence of schizophrenia. Third, our sequencing design focused solely on protein-coding regions, so we did not analyze potentially pathogenic variants in promoter regions, deep intronic regions, or structural regulatory boundaries. Fourth, due to the extreme rarity of the *HSPA1L*^p.Val262Met^ variant, we were unable to access multi-generational family pedigrees to confirm familial segregation with the disease phenotype. Finally, when studies with limited sample sizes detect statistically significant associations for ultra-rare variants, such as *HSPA1L*^p.Val262Met^, the estimated effect size (OR = 37.65) is likely highly inflated. We should be cautious, as while the association is present in this cohort, the true effect size is likely much smaller. Therefore, independent validation in a significantly larger cohort is essential.

## 5. Conclusions

In conclusion, while common variants in the *HSPA1A*, *HSPA1B*, and *HSPA1L* genes do not seem to be significant genetic risk factors for schizophrenia in this cohort, the ultra-rare mutation, *HSPA1L*^p.Val262Met^, significantly reduces protein expression. These preliminary findings suggest that a potential loss-of-function or reduced expression of the *HSPA1L* gene may be a predisposing factor contributing to schizophrenia vulnerability in certain individuals, though independent replication and further in vivo models are necessary to definitively establish causality. However, these findings underscore the importance of investigating rare, high-impact mutations that may be overlooked in large-scale association studies, as they can provide crucial insights into the molecular mechanisms underlying the disease. To validate our initial findings and to further assess the influence of rare mutations in the *HSPA1L* gene on the risk and psychopathology of schizophrenia, larger studies using independent sample sets are required. Additionally, in vitro and in vivo functional studies should be considered the next critical step in exploring the regulatory effects of *HSPA1L*^p.Val262Met^ mutation in neural circuits.

## Figures and Tables

**Table 1:** Bioinformatic analysis of coding variants of the *HSPA1A*, *HSPA1B*, and *HSPA1L* genes identified in 100 patients with schizophrenia.

*Variant (Amino Acid Change)*	*dbSNP ID*	*MAF*			*In Silico Analysis for Amino Acid Substitution*		
		1000G	gnomAD	*Taiwan Biobank*	*Polyphen-2*	*SIFT*	*CADD* *Raw/PHRED*
*HSPA1A*							
c.222T>C (p.Ile74=)	rs1043620	0.0367	0.1206	0.0083	N/A	N/A	N/A
c.330G>C (p.Glu110Asp)	rs562047	0.2282	0.1253	N/R	Benign	Deleterious	2.69/22.9
c.1695G>C (p.Ala565=)	rs506770	0.1532	0.2137	0.2219	N/A	N/A	N/A
*HSPA1B*							
c.330G>C (p.Glu110Asp)	rs144223778	0.0709	0.0494	0.1006	Benign	Deleterious	2.93/23.4
c.1053G>A (p.Gln351=)	rs754888705	N/R	0.4485	N/R	N/A	N/A	N/A
c.1461C>T (p.Asn487=)	rs1373025992	N/R	0.0001	N/R	N/A	N/A	N/A
c.1695G>C (p.Ala565=)	rs56280220	0.0160	0.0129	N/R	N/A	N/A	N/A
c.1710T>G (p.Val570=)	rs35682610	N/R	0.0043	N/R	N/A	N/A	N/A
c.1860C>G (p.Gly620=)	rs539689	0.4265	0.4882	0.3727	N/A	N/A	N/A
*HSPA1L*							
c.22G>C (p.Ala8Pro)	rs9469057	0.0128	0.0054	0.0292	Probably damaging	Deleterious	2.65/22.8
c.22G>A (p.Ala8Thr)	rs9469057	N/R	0.0001	0.0121	Probably damaging	Deleterious	2.66/22.9
c.784G>A (p.Val262Met)	rs142416335	N/R	<0.0001	N/R	Possibly damaging	Tolerated	2.79/23.1
c.919T>C (p.Leu307=)	rs35326839	0.0347	0.0060	0.1153	N/A	N/A	N/A
c.1221G>A (p.Thr407=)	rs2075799	0.1536	0.0844	0.1779	N/A	N/A	N/A
c.1478C>T (p.Thr493Met)	rs2227956	0.1228	0.1635	0.1823	Benign	Tolerated	1.08/13.98
c.1673A>C (p.Glu558Ala)	rs2227955	0.0483	0.0296	0.0047	Benign	Tolerated	2.56/22.7
c.1804G>A (p.Glu602Lys)	rs2075800	0.2552	0.3113	0.3924	Possibly damaging	Deleterious	3.83/26.4

Reference version: GRCh37; *HSPA1A*: NM_005345.6; *HSPA1B*: NM_005346.6; *HSPA1L*: NM_005527.4; N/R: not registered; N/A: not available; MAF: minor allele frequency.

**Table 2:** Genotype and allele frequencies of four SNPs of the *HSPA1L* gene in patients with schizophrenia (SZ) and healthy controls from Taiwan Biobank (Ctrl).

Variant	Type	*N*	Genotype			HWE p	*p*	Allele		*p*	Allelic OR	95% CI
c.22G>C			G/G	G/C	C/C			G	C			
p.Ala8Pro	SZ	475	453	22	0	0.6054	0.5103	928	22	0.4223	0.80	0.50~1.29
rs9469057	Ctrl	1479	1397	79	3	0.0975		2873	85			
c.22G>A			G/G	G/A	A/A			G	A			
p.Ala8Thr	SZ	470	453	17	0	0.6900	0.2066	923	17	0.1631	1.51	0.84~2.70
rs9469057	Ctrl	1492	1397	36	0	0.6302		2948	36			
c.784G>A			G/G	G/A	A/A			G	A			
p.Val262Met	SZ	518	512	6	0	0.8945	<0.0001	1030	6	<0.0001	37.65	2.12~668.47
rs142416335	Ctrl	1492	1492	0	0	1.0000		2984	0			
c.1804G>A			G/G	G/A	A/A			G	A			
p.Glu602Lys	SZ	519	179	269	71	0.0571	0.2485	627	411	0.8412	1.01	0.88~1.17
rs2075800	Ctrl	1492	551	711	230	0.9797		1813	1171			

OR: odds ratios; CI: confidence intervals.

## Data Availability

The raw data are available upon request of the corresponding author.

## Supplementary Materials

The following supporting information can be downloaded at: https://www.mdpi.com/article/10.3390/genes17070727/s1, Supplementary Figure S1: Sequence electropherograms of the *HSPA1L*^p.Val262Met^ mutation; Supplementary Table S1: Primer sequences, optimal annealing temperature (Tm) and size of PCR products of the *HSPA1A*, *HSPA1B*, *HSPA1L* gene.

## References

[1] Owen M.J., Sawa A., Mortensen P.B. (2016). Schizophrenia. Lancet.

[2] Kavanagh D.H., Tansey K.E., O’Donovan M.C., Owen M.J. (2015). Schizophrenia genetics: Emerging themes for a complex disorder. Mol. Psychiatry.

[3] Howes O.D., Onwordi E.C. (2023). The synaptic hypothesis of schizophrenia version III: A master mechanism. Mol. Psychiatry.

[4] Müller N. (2018). Inflammation in schizophrenia: Pathogenetic aspects and therapeutic considerations. Schizophr. Bull..

[5] Daugaard M., Rohde M., Jäättelä M. (2007). The heat shock protein 70 family: Highly homologous proteins with overlapping and distinct functions. FEBS Lett..

[6] Kiang J.G., Tsokos G.C. (1998). Heat shock protein 70 kDa: Molecular biology, biochemistry, and physiology. Pharmacol. Ther..

[7] Solarz A., Majcher-Maślanka I., Kryst J., Chocyk A. (2021). A search for biomarkers of early-life stress-related psychopathology: Focus on 70-kDa heat shock proteins. Neuroscience.

[8] Radons J. (2016). The human HSP70 family of chaperones: Where do we stand?. Cell Stress Chaperones.

[9] Karunanithi S., Barclay J.W., Brown I.R., Robertson R.M., Atwood H.L. (2002). Enhancement of presynaptic performance in transgenic Drosophila overexpressing heat shock protein Hsp70. Synapse.

[10] Bechtold D.A., Rush S.J., Brown I.R. (2000). Localization of the heat-shock protein Hsp70 to the synapse following hyperthermic stress in the brain. J. Neurochem..

[11] Zatsepina O.G., Evgen’ev M.B., Garbuz D.G. (2021). Role of a heat shock transcription factor and the major heat shock protein Hsp70 in memory formation and neuroprotection. Cells.

[12] Bates P.R., Hawkins A., Mahadik S.P., McGrath J.J. (1996). Heat stress lipids and schizophrenia. Prostaglandins Leukot. Essent. Fat. Acids.

[13] Bamne M., Wood J., Chowdari K., Watson A.M., Celik C., Mansour H., Klei L., Gur R.C., Bradford L.D., Calkins M.E. (2012). Evaluation of HLA polymorphisms in relation to schizophrenia risk and infectious exposure. Schizophr. Bull..

[14] Kodavali C.V., Watson A.M., Prasad K.M., Celik C., Mansour H., Yolken R.H., Nimgaonkar V.L. (2014). HLA associations in schizophrenia: Are we re-discovering the wheel?. Am. J. Med. Genet. B Neuropsychiatr. Genet..

[15] Kim J.J., Lee S.J., Toh K.Y., Lee C.U., Lee C., Paik I.H. (2001). Identification of antibodies to heat shock proteins 90 kDa and 70 kDa in patients with schizophrenia. Schizophr. Res..

[16] Schwarz M.J., Riedel M., Gruber R., Ackenheil M., Müller N. (1999). Antibodies to heat shock proteins in schizophrenic patients: Implications for the mechanism of the disease. Am. J. Psychiatry.

[17] Kowalczyk M., Kucia K., Owczarek A., Suchanek-Raif R., Merk W., Paul-Samojedny M., Kowalski J. (2018). Association studies of *HSPA1A* and *HSPA1L* gene polymorphisms with schizophrenia. Arch. Med. Res..

[18] Kim J.J., Mandelli L., Lim S., Lim H.K., Kwon O.J., Pae C.U., Serretti A., Nimgaonkar V.L., Paik I.H., Jun T.Y. (2008). Association analysis of heat shock protein 70 gene polymorphisms in schizophrenia. Eur. Arch. Psychiatry Clin. Neurosci..

[19] Kowalczyk M., Kucia K., Owczarek A., Suchanek-Raif R., Merk W., Fila-Danilow A., Paul-Samojedny M., Choreza P., Kowalski J. (2020). Association of *HSPA1B* polymorphisms with paranoid schizophrenia in a Polish population. Neuromolecular Med..

[20] Pae C.U., Drago A., Kim J.J., Mandelli L., De Ronchi D., Serretti A. (2009). The impact of heat shock protein 70 gene variations on clinical presentation and outcome in schizophrenic inpatients. Neuropsychobiology.

[21] Pae C.U., Kim T.S., Kwon O.J., Artioli P., Serretti A., Lee C.U., Lee S.J., Lee C., Paik I.H., Kim J.J. (2005). Polymorphisms of heat shock protein 70 gene (*HSPA1A*, *HSPA1B* and *HSPA1L*) and schizophrenia. Neurosci. Res..

[22] Kowalczyk M., Owczarek A., Suchanek R., Paul-Samojedny M., Fila-Danilow A., Borkowska P., Kucia K., Kowalski J. (2014). Heat shock protein 70 gene polymorphisms are associated with paranoid schizophrenia in the Polish population. Cell Stress Chaperones.

[23] Purcell S.M., Moran J.L., Fromer M., Ruderfer D., Solovieff N., Roussos P., O’Dushlaine C., Chambert K., Bergen S.E., Kahler A. (2014). A polygenic burden of rare disruptive mutations in schizophrenia. Nature.

[24] Fromer M., Pocklington A.J., Kavanagh D.H., Williams H.J., Dwyer S., Gormley P., Georgieva L., Rees E., Palta P., Ruderfer D.M. (2014). De novo mutations in schizophrenia implicate synaptic networks. Nature.

[25] Wang Y.Y., Hsu S.H., Tsai H.Y., Cheng F.Y., Cheng M.C. (2022). Transcriptomic and proteomic analysis of CRISPR/Cas9-mediated *ARC*-knockout HEK293 cells. Int. J. Mol. Sci..

[26] Craddock N., O’Donovan M.C., Owen M.J. (2007). Phenotypic and genetic complexity of psychosis. Invited commentary on ... Schizophrenia: A common disease caused by multiple rare alleles. Br. J. Psychiatry.

[27] McClellan J.M., Susser E., King M.-C. (2007). Schizophrenia: A common disease caused by multiple rare alleles. Br. J. Psychiatry.

[28] Schork N.J., Murray S.S., Frazer K.A., Topol E.J. (2009). Common vs. rare allele hypotheses for complex diseases. Curr. Opin. Genet. Dev..

[29] Manolio T.A., Collins F.S., Cox N.J., Goldstein D.B., Hindorff L.A., Hunter D.J., McCarthy M.I., Ramos E.M., Cardon L.R., Chakravarti A. (2009). Finding the missing heritability of complex diseases. Nature.

[30] Colhoun H.M., McKeigue P.M., Davey Smith G. (2003). Problems of reporting genetic associations with complex outcomes. Lancet.

[31] Yu W.W., Cao S.N., Zang C.X., Wang L., Yang H.Y., Bao X.Q., Zhang D. (2018). Heat shock protein 70 suppresses neuroinflammation induced by α-synuclein in astrocytes. Mol. Cell Neurosci..

[32] Wisniewska M., Karlberg T., Lehtiö L., Johansson I., Kotenyova T., Moche M., Schüler H. (2010). Crystal structures of the ATPase domains of four human Hsp70 isoforms: HSPA1L/Hsp70-hom, HSPA2/Hsp70-2, HSPA6/Hsp70B’, and HSPA5/BiP/GRP78. PLoS ONE.

