# Supplementary material for: Genetic Analysis of the HSPA1A, HSPA1B, and HSPA1L Genes in Patients with Schizophrenia from Taiwan
Source: Genes (Basel). 2026 Jun 23;17(7):727. doi: 10.3390/genes17070727 (PMC13408748; doi:10.3390/genes17070727)
Supplement: Supplementary file 1 [file genes-17-00727-s001.zip › Supplemenatry Table S1.pdf]

Supplementary Table S1. Primer sequences, optimal annealing temperature (Tm) and size of PCR products of the *HSPA1A*, *HSPA1B*, *HSPA1L* gene.

Target	PCR Forward	PCR Reverse	Size (bp)	Tm (°C)
HSPA1A	5`-tgattggctcagaagggaaa-3`	5`-gaccctactgacccccaaat-3`	2511	60
HSPA1B	5`-attggctcagaaggggaaag-3`	5`-tatccctccgcaaatatcca-3`	2632	60
HSPA1L	5`- gctggagcctaggagatcaa-3`	5`- cacacacacacacacaaaatgag-3`	2552	60
