# Supplementary figures and images for: Genetic Analysis of the HSPA1A, HSPA1B, and HSPA1L Genes in Patients with Schizophrenia from Taiwan
Source: Genes (Basel). 2026 Jun 23;17(7):727. doi: 10.3390/genes17070727 (PMC13408748; doi:10.3390/genes17070727)

**Supplementary Figure S1.** Sequence electropherograms of the *HSPA1L*<sup>p.Val262Met</sup> mutation.

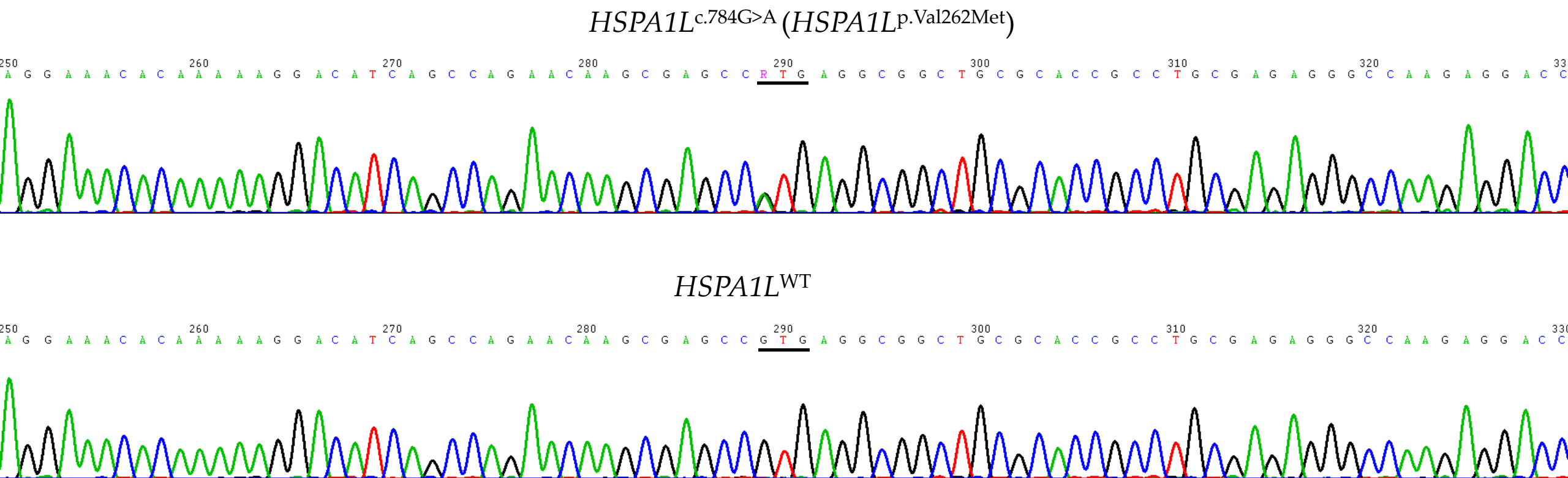

Supplement: Supplementary file 1 [file genes-17-00727-s001.zip › Supplementary Figure S1.pdf]
